# Parkinson’s disease updates: Addressing the pathophysiology, risk factors, genetics, diagnosis, along with the medical and surgical treatment

**DOI:** 10.1097/MS9.0000000000001142

**Published:** 2023-08-08

**Authors:** Priyadarshi Prajjwal, Herson S Flores Sanga, Kirtish Acharya, Tamara Tango, Jobby John, Rene S.C. Rodriguez, Mohammed Dheyaa Marsool Marsool, Mukhamed Sulaimanov, Aneeqa Ahmed, Omniat A. Hussin

**Affiliations:** a Bharati Vidyapeeth Deemed University, Pune, Maharashtra; b Shadan Hospital and Institute of Medical Sciences, Hyderabad, Telangana; c Maharaja Krishna Chandra Gajapati Medical College and Hospital, Brahmapur, Odisha; d Dr. Somervell Memorial CSI Medical College and Hospital, Neyyāttinkara, Kerala, India; e Faculty of Medicine Universitas, Jakarta, Indonesia; f Universidad de Guadalajara, Mexico; g University of Baghdad/Al-Kindy College of Medicine, Baghdad, Iraq; h Kyrgyz State Medical Academy, Bishkek, Kyrgyzstan; i Department of Telemedicine, Hospital Nacional Carlos Alberto Seguin Escobedo, Arequipa, Peru; j Department of Medicine, Sudan Academy of Sciences, Khartoum, Sudan

**Keywords:** diagnosis, genetics, orthostatic hypotension, Parkinson’s disease, pathophysiology, treatment

## Abstract

After only Alzheimer’s disease (AD), Parkinson’s disease (PD) is the second most prevalent neurodegenerative disease. The incidence of this disease increases with age, especially for those above 70 years old. There are many risk factors that are well-established in the contribution to the development of PD, such as age, gender, ethnicity, rapid eye movement sleep disorder, high consumption of dairy products, traumatic brain injury, genetics, and pesticides/herbicides. Interestingly, smoking, consumption of caffeine, and physical activities are the protective factors of PD. A deficiency of dopamine in the substantia nigra of the brainstem is the main pathology. This, subsequently, alters the neurotransmitter, causing an imbalance between excitatory and inhibitory signals. In addition, genetics is also involved in the pathogenesis of the disease. As a result, patients exhibit characteristic motor symptoms such as tremors, stiffness, bradykinesia, and postural instability, along with non-motor symptoms, including dementia, urinary incontinence, sleeping disturbances, and orthostatic hypotension. PD may resemble other diseases; therefore, it is important to pay attention to the diagnosis criteria. Parkinson’s disease dementia can share common features with AD; this can include behavioral as well as psychiatric symptoms, in addition to the pathology being protein aggregate accumulation in the brain. For PD management, the administration of pharmacological treatment depends on the motor symptoms experienced by the patients. Non-pharmacological treatment plays a role as adjuvant therapy, while surgical management is indicated in chronic cases. This paper aims to review the etiology, risk factors, protective factors, pathophysiology, signs and symptoms, associated conditions, and management of PD.

## Introduction

Highlights

- Parkinson’s disease (PD) is a neurological disorder characterized by the degeneration of dopamine-producing neurons in the brain, leading to motor symptoms such as tremors, stiffness, slowness of movement, and balance difficulties.
- The exact cause of PD is unknown, but it is believed to be influenced by genetics, age-related changes in neurons, and the presence of abnormal proteins called Lewy bodies.
- PD is associated with various underlying mechanisms, including oxidative stress, mitochondrial damage, protein misfolding, excitotoxicity, inflammation, and autoimmune responses.
- Treatment of PD aims to alleviate symptoms through medications like levodopa and dopamine agonists, as well as surgical interventions like deep brain stimulation. However, current treatments do not slow down or halt the progression of the disease.
- PD management may involve nondrug therapies, exercise therapies, psychological interventions, and surgical options when medical treatment becomes insufficient. Other diseases can complicate PD management, and the development of dementia is also associated with PD.

Parkinson’s disease (PD) is a neurodegenerative condition that largely affects the motor system. Tremors, stiffness, and bradykinesia are some of the symptoms. Yet, typical non-motor signs of PD include dementia and cognitive decline. The quality of life and independence of the patient may be impacted by these cognitive deficits, which can vary from mild cognitive impairment to dementia^1^.

A kind of dementia known as PD dementia (PDD) is thought to affect between 50 and 80% of people with PD. Although the etiology of PDD is complicated and not entirely understood, it is believed to be caused by the buildup of aberrant protein aggregates in the brain, such as alpha-synuclein. Cognitive deterioration can ensue from these aggregates, which can cause neuronal malfunction and death. In addition, there may be a role for inflammation and oxidative stress in the development of PDD^2,3^.

Age, the length of the disease, the severity of the motor impairment, and the existence of certain genetic variations are all risk factors for the development of PDD. PDD and other neurodegenerative conditions, including Parkinson’s plus syndromes, Alzheimer’s dementia, and Lewy body dementia may also be related^1,2^.

Despite significant advancements in our understanding of PD, there are still gaps in our knowledge regarding the precise risk factors contributing to its development, the underlying pathophysiological mechanisms, and the optimal management strategies for both motor and non-motor symptoms (NMSs). This review article aims to address these gaps by providing an up-to-date overview of the current literature on the etiology, pathophysiology, and management of PD. By synthesizing the existing knowledge, this review seeks to enhance our understanding of PD and guide future research efforts to improve the prevention, diagnosis, and treatment of this debilitating condition.

### Methodology

A systematic search was conducted to identify relevant articles for this narrative review. The following databases were searched: PubMed, Medline, Scopus, and Web of Science. The search terms used included ‘Parkinson’s disease’, ‘genetics’, ‘pathophysiology’, ‘diagnosis’, ‘treatment’, and ‘orthostatic hypotension’ using a combination of AND/OR. The search was limited to articles published in English.

The search strategy involved a combination of keywords and MeSH (Medical Subject Headings) terms to ensure comprehensive coverage of the literature. The initial search yielded a large number of articles, which were then screened based on their titles and abstracts for relevance. Full-text articles were retrieved for further evaluation, and additional relevant studies were identified through a manual search of the reference lists of included articles.

The selected articles were critically reviewed and analyzed to extract key information on the etiology, risk factors, pathophysiology, diagnosis, and treatment of PD. Data synthesis was performed to present a comprehensive overview of the current understanding in these areas. The limitations and gaps in the existing literature were also identified and discussed at the end of this review.

## Etiology

Second only to Alzheimer’s disease (AD), PD has been recognized as the most common disorder causing neuronal degeneration^1,3^. The etiology of which is not well understood. It is an idiopathic, multifactorial disease in which genetic and environmental factors interplay^4^. Mitochondrial dysfunction, glial cell excitation, alpha-synuclein (αSyn) deposition, and calcium channel abnormalities seen in PD are all the result of the oxidative damage caused by free radicals’ formation^5^.

## Risk factors

In the United States, almost a million people have PD (U.S.). The 2022 Parkinson’s Foundation-sponsored research found that almost 90 000 individuals in the United States are diagnosed with PD each year. This new incidence is 1.5 times higher compared to the PD incidence rate of 40 000–60 000 per year^6^. In North America, the increase in PD incidence aligns with the growth of the aging population. The incidence rates are higher in certain geographic areas, such as the northeastern and midwestern U.S. PD occurred at a rate of 108–212/100 000 person-years in individuals aged 65 and over, 162–277 in males, and 66–161 in women.

PD was found in 47–77 individuals out of every 100 000 aged 45 and over^6^. It has a prevalence ranging from 41 every 100 000 in the fourth decade of one’s life to over 1900 per 100 00 in individuals over the age of 80^7^. And this incidence tends to increase as individuals grow older. The prevalence of PD climbed from less than 1% of men and women aged 45–54 years to 4% of men and 2% of women aged 85 and older, according to a meta-analysis of four North American populations.

### Age

The most significant risk factor for PD is age. And the average life expectancy is 70, which is becoming more prevalent as people become older. A minority have the illness starting before the age of 50.

### Gender

Gender is a well-known risk factor, as the ratio between males and females is around 3:2^8^. Women have minimal benefits over males since the prevalence of the disease is lower in the 50–59 age range and their age at onset is older^9^. Males were disabled for a longer period of time^10^.

### Ethnicity

Ethnicity is considered one risk factor for PD. In the U.S., Hispanic-ethnic origin has the highest incidence of PD followed by non-Hispanic Whites, Asians, and Blacks^11^.

### Rapid eye movement (REM) sleep disorder

One risk factor for PD is disorders affecting sleep behavior, REM Sleep Behavior Disorder (RBD). Everyone with RBD will not get PD, yet, RBD is one of the first signs of PD in some patients.

### Dairy products

Increased milk and dairy consumption are more likely resulting into the development of PD. This has been demonstrated in studies such as the Nurses’ Health Study and the Health Professionals Follow-up Study (HPFS), the Honolulu-Asian Ageing Study (HAAS), and the Cancer Prevention Study II Nutrition (CPS-IIN)^12,13^. These cohorts showed a meta-analysis relative risk (RR) of PD of 1.6 (*P*=0.0001) when the greatest and lowest dairy consumption categories were compared^13^. The Finnish Mobile Clinic (FMC) cohort found a link between PD and milk intake^14^.

In the HAAS cohort, heptachlor epoxide residues were more abundant in the brains of individuals who drank more milk than those who did not, suggesting that the pollutant may be linked to PD risk^15^. While the possibility of contaminated milk underlying the disease risk association cannot be ruled out, the cumulative evidence from several cohorts is more consistent with the risk of PD being associated with dairy products’ urate-lowering actions^16^.

### Traumatic brain injury

Blood–brain barrier (BBB) can get dysfunctional by traumatic brain injury (TBI). TBI not only results in the disruption of the BBB, but in addition to that, it results in inflammation of the brain, dysfunction of the mitochondria, release of glutamate increases, and deposition of αSyn. All of these processes can have an effect on increasing the incidence of PD^17^. Over 13 000 PD patients were assessed in a study, the results of which showed that after a concussion, the RR of PD was 6.6 within 3 months, 1.9 between 4 and 12 months, 1.8 between 1 and 4 years, 1.4 between 5 and 9 years, and no significant increase in the risk of PD after 10 years^18^.

The RR for PD was 3.34 within 12 months following hospitalization for a head injury in a study of nearly 18 000 PD patients but declined to 1.28 in years 1–4, 1.18 in years 5–9, and 1.17 after 10 years^19^. Both trials found an early increase in PD risk owing to frequent falls, but it is difficult to determine if there is a long-term increase in PD^20^.

### Genetics

Although family history is seen in 10–15% of patients, and 5% exhibit Mendelian inheritance, PD in general, is an idiopathic disorder^4,21^. *PARK* genes are the ones that cause PD, and 23 of them have been related to the disease^22^. *PARK* gene mutations have an autosomal dominant or autosomal recessive inheritance pattern. Mutations in the lysosomal enzyme responsible for the hydrolysis of glucocerebrosides GBA1 (β-glucocerebrosidase) are the most common genetic risk factors for PD^23^. Gaucher disease is caused by GBA1 mutations^24^. Others include MHC class II (HLA-DQB1) and the MAPT (microtubule-associated protein tau) gene, which encodes the protein tau^25,26^.

#### Autosomal dominant PD

This is a PD type induced by the α-synuclein gene *(SNCA)* point mutation^27^. The most common autosomal dominant monogenic PD is caused by alterations to the gene expressing leucine-rich repeat kinase 2 *(LRRK2)*. *LRRK2* contains six pathogenic mutations, the most common of which is p.G2019S, which accounts for around 1% of sporadic and 4% of familial PD worldwide^27,28^.

#### Autosomal recessive PD

When compared to conventional PD, it manifests early. This recessive variant is caused by three *PARK* genes that have been connected to mitochondrial homeostasis *(PRKN, PINK1*, and *DJ1)*. The *PRKN* gene encodes the mitochondrial proteins’ quality control which are the *PINK1* and parkin. *PINK1* recruiting parkin to faulty mitochondria promotes mitophagy^28^. *PRKN* mutations are the most prevalent cause of autosomal recessive familial PD^29^.

The genes involved in PD are described in Table 1.

**Table 1:** Genes involved in monogenic Parkinson’s disease.

Gene	Mode of inheritance	Frequency
*SNCA*	AD	Rare, with recent investigations showing a frequency ranging from 0.045 to 1.1%^30^
*LRRK2*	AD	1% of PD
*VPS35*	AD	Rare
*PRKN*	AR	The most common cause of EOPD (12.5% of recessive PD)^31^
*PINK1*	AR	The second leading cause of EOPD (1.9% of recessive PD)^31^.
*PARK7* (*DJ1*)	AR	0.16% of recessive PD^31^
*TAF1*	X-linked	0.34 per 100 000 in the Philippines, Island of Panay 5.24 per 100 000
*ATP13A2*	AR	Rare^32^
*DCTN1*	AD	Rare^32^
*DNAJC6*	AR	Rare^32^
*FBXO7*	AR	Rare^32^
*PLA2G6*	AR	Rare^33^
*SYNJ1*	AR	Rare
*CHCHD2*	AD	Rare
*LRP10*	AD	Rare
*TMEM230*	AD	Rare
*UQCRC1*	AD	Rare
*VPS13C*	AR	Rare

AD, autosomal dominant; AR, autosomal recessive; EOPD, early-onset Parkinson’s disease; PD, Parkinson’s disease.

### Pesticides/herbicides

In 1983, it was discovered that there was an association between nigrostriatal degeneration and the chemical 1-methyl-4-phenyl-1,2,3,6-tetrahydropyridine (MPTP) when many persons acquired characteristic PD symptoms after taking a medication tainted with MPTP. The neurotoxin MPP+ (1-methyl-4-phenylpyridinium) is an inhibitor of mitochondrial complex-I that, in the substantia nigra, specifically destroys dopaminergic cells^34,35^. It was found that there is a link between pesticides and PD by some studies. And it was found that there is a link between professional pesticide exposure and late-onset PD in males, with an odds ratio (OR) of 2.2 in a case–control study^36^. Rotenone, a pesticide, is also a selective inhibitor of complex-I that promotes the depletion of dopamine in animal models of PD^37,38^. Numerous epidemiological studies have looked at the relationship between exposure to these chemicals and the chance of developing PD^39^.

## Protective factors

### Smoking

Numerous researches on current and previous smokers have found that cigarette smoking may serve a protective effect in PD. PD and both intensity and length of smoking correlate inversely, which is more prominent among current smokers than former smokers. When the number of years following smoking grows, so does the level of protection^40–48^. Despite the fact that cigarettes contain hundreds of compounds, nicotine has a protective effect because it activates striatal dopamine neurons, which are destroyed in PD. It was also found that neurons in animal models were protected from the effects of nicotine^49–52^. This inverse relation between smoking and PD has been explored in over 40 investigations undertaken by different investigators over the last 50 years. Smoking has a dose-dependent impact that is proportional to the number of cigarette pack years^49–52^. One study found that the decrease in incidence does not increase with age, implying that smoking delays PD^53^. A recent research on twins found that smoking lowered the chance of PD, and the authors concluded that smoking has a real protective effect^54^.

### Coffee

Caffeine intake is believed to account for over half of the world’s adult population. This is somewhat because of its effects on cognition and also because it is a psychostimulant, which reduces weariness and improves performance^55^. Coffee consumption reduces the RR of PD by 0.5–0.8, with a dose-dependent impact reported in some studies^20^. The Honolulu Heart Program, a large prospective study in which 8004 Japanese-American men were studied at 30 years of follow-up, discovered that coffee consumption on a daily basis after an age and smoking adjustments – an intake of at least 784 mg/kg throughout midlife – decreases the chance of getting PD at age 65 by five-fold compared to non-coffee users^56^.

The Health Professionals’ Follow-Up Study and the Nurses’ Health Study, which included 47 351 men and 88 565 women, as well as the Finnish Mobile Clinic Health Examination Survey, NeuroGenetics Research Consortium, and Danish case–control study involving idiopathic PD^45,48,57^, all supported the inverse relationship between caffeine consumption and the development of PD.

A meta-analysis of 13 trials comprising 901 764 people indicated a connection between caffeine consumption and PD risk, with a maximal preventive impact of roughly 3 cups/day^58^. Caffeine intake is related with a lower incidence of PD, according to a systematic review of 120 observational studies^45,56,59–61^. It has no discernible deleterious impact on cardiac function, bone health, or the occurrence of cancer^62–67^. According to the Health Professional Follow-up Study, males and caffeinated coffee had a stronger inverse connection than women^59–61^.

A cohort in 2012 showed that the opposite relationship is strong among postmenopausal women who have never undergone hormone replacement treatment^68^. As a result, the combination of coffee and estrogen can alter the chance of developing PD. A case–control study was conducted among 566 subjects, including idiopathic PD patients and healthy controls, and lower coffee consumption and plasma urate levels were found to be inversely related to idiopathic PD^69^.

### Physical activity

It is an essential lifestyle component that may influence the incidence, severity, and progression of PD in physical status^70^. Strength exercises, aerobics, and other forms of physical activity during midlife are linked to decreased PD risks, a better disease prognosis, and fewer major consequences^71–73^. A major worldwide multicenter cohort research on early PD found that younger people and men had higher self-reported activity scores. Inactivity and its effects may be more dangerous for older individuals and for women^74^.

### Statin

A cohort research that was in statin-free adults found that there’s a link between the levels of cholesterol and the PD risk. Total cholesterol levels larger than 180 mg/dl and low-density lipoprotein cholesterol levels greater than 110 mg/dl were shown to be related with a decreased risk of PD in middle-aged men and older women^75^. Men have a lower chance of PD, while women do not. This investigation was unable to determine the link between cholesterol and PD^70^. Lin and colleagues conducted a prospective research to examine the relationship between statin dose and PD incidence among diabetic patients in Taiwan and the adjusted hazard ratios (HRs) of statin usage for PD incidence reduced in both males and females^76,77^.

Except for lovastatin, statins had a dose-dependent protective impact on PD incidence^76,77^. A meta-analysis of observational studies revealed that the pooled RR of statin usage for PD reduced, although there was variability across estimates^78^. It was observed that the pooled odds ratio (OR) of statin usage for PD reduced, with atorvastatin showing the greatest reduction in PD risk^79^. Further research is needed to confirm the preventive benefit of statins for PD.

### Urate

Urate is a purine metabolite that occurs within the human body. It is anti-oxidant and neuroprotective^70,80,81^. Higher levels of urate have been linked to a decreased risk and slower course of idiopathic PD^70,80,81^. Increased urate levels were related with a lower prevalence and slower PD coin males but not in women^82,83^. Urate appears to have neuroprotective effects in men due to its ability to modify intrinsic connectivity networks, which reflect spontaneous neural activity and give information indirectly about the functional status of the brain^84^.

## Pathophysiology of PD and dementia

**Figure 1:**
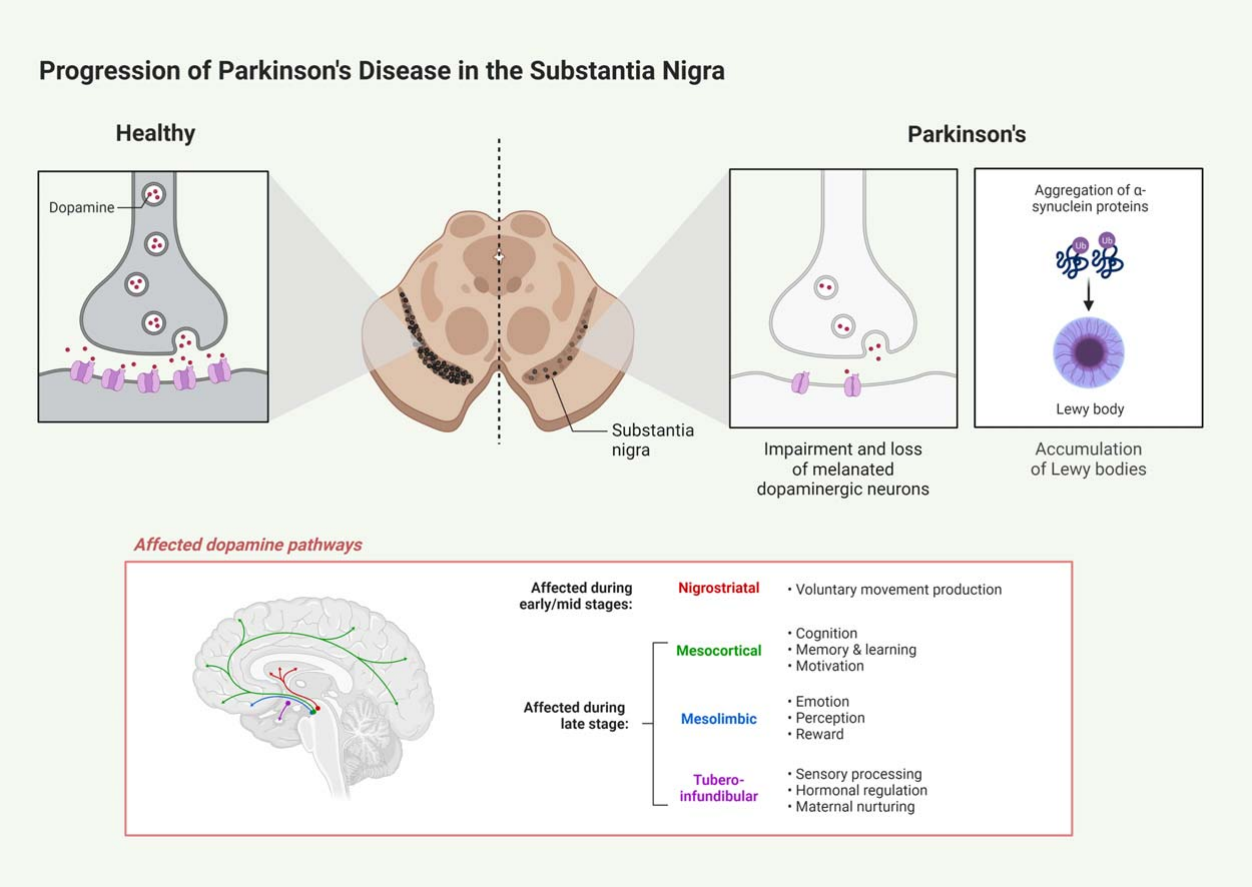
Progression of Parkinson’s disease in the substantia nigra. The pathophysiology involves the loss of dopaminergic neurons and Lewy bodies, which are aggregations of alpha-synuclein protein clusters bound to ubiquitin. This can damage the dopaminergic neurons when they form in the substantia nigra and interfere with pathways responsible for motor function and other cognitive processes.

PD pathophysiology is a complex process that requires a detailed analysis to understand and treat the condition effectively. PD is defined as a neurological disorder caused by dopamine-producing neurons degeneration in the brain, which leads to a characteristic set of motor symptoms. These can include tremors, stiffness, movement slowness, and difficulty achieving balance. While the precise PD etiology is unknown, it is thought to be linked to genetics, age-related changes in neurons, and issues with specific proteins known as Lewy bodies (Fig. 1)^85,86^. PD can significantly impair the patient’s motor and speech abilities. Parkinson’s primarily affects the dopamine-producing neurons in the brain, which control movement; these nerve cells become impaired or die over time. As a result, the person’s movements become limited, slow, and are sometimes accompanied by tremors. The progression of PD symptoms depends on several factors, including the individual’s age and corresponding brain changes.

The main pathological cause of PD is a dopamine deficiency within the brain. The dopamine-producing neurons in the brain are typically located in an area called the substantia nigra. Dysfunction of these cells, either through death or their inability to produce and secrete dopamine, will decrease dopamine concentration in the brain^20^. This is due to the fact that dopamine in the substantia nigra is normally transferred to the basal ganglia, the brain area that is responsible for controlling movement.

Many processes have been hypothesized to explain why dopamine-producing neurons in the substantia nigra become impaired or die in people with PD. A commonly accepted theory is that PD is caused by an accumulation of misfolded proteins. These misfolded proteins lead to oxidative stress and mitochondrial damage, which, over time, leads to cell death. Another proposed mechanism suggests that PD is caused by an autoimmune response, in which the immune system mistakenly identifies its own tissues as ‘foreign’ and attempts to attack them^87^.

The classic motor symptoms of PD, such as rigidity, tremors, and bradykinesia, are due to basal ganglia dysfunction arising from a decrease in the activity of dopamine-responsive neurons in the substantia nigra^88–90^. Dopamine is an inhibitory neurotransmitter that is essential to basal ganglia functioning, and a decrease in its activity is thought to result from a progressive substantia nigra dopaminergic neuronal loss in the pars compacta. This neuronal degeneration is the result of oxidative stress, mitochondrial dysfunction, disturbance of protein folding, excitotoxicity, and inflammation, which eventually lead to apoptotic death of the neurons^91^.

In the substantia nigra dopaminergic neuron loss affects other neurotransmitter systems as well, such as the glutamate-producing neurons in the striatum. These changed neurotransmitter networks resulted in an imbalance of excitatory and inhibitory impulses in the basal ganglia, leading to basal ganglia dysfunction and characteristic PD motor symptoms^92,93^. NMSs of PD, in addition to motor symptoms, are related with disruptions in other neurotransmitter systems, as well as the autonomic and endocrine systems, vascular systems, and beyond. A reduction in dopamine, acetylcholine, and noradrenaline, for example, causes alterations in mood, sleep–wake cycle, fluid control, and other areas. Changes in the vascular system can lead to problems with cognition and memory. These NMSs can even occur without the motor symptoms for some individuals, leading to a more complex picture of the disease^94^.

The pathophysiology of PD is complex and highly variable, and these underlying mechanisms can lead to a wide variety of clinical symptoms and different degrees of severity. In recent years, the focus of research on PD has shifted toward a more holistic understanding of the disease, taking into account the various physiological systems and pathways that are affected by this neurodegenerative disorder. This will help in developing treatments that can target multiple symptoms, as well as improving our understanding of the pathophysiology of this devastating neurological disorder^95,96^.

In addition to dopamine deficiency, PD is often accompanied by several other changes in the brain. For example, it is almost always accompanied by a decrease in the size of the striatum in addition to a decrease in glial cell numbers. The striatum is involved in the movement and reward processing, while glial cells are responsible for providing support and protection to neurons. It is important to note that these changes are usually evident only in the advanced stages of the disorder^97^.

To treat Parkinson’s effectively, it is important to understand the pathophysiology involved. While researchers are still uncovering the mechanisms behind the disorder, current treatments focus on replacing the lost dopamine with medications such as levodopa^98–100^, which is broken down into dopamine in the brain. Dopamine agonists, such as pramipexole and ropinirole, are also used to stimulate the brain’s remaining dopamine receptors and thus enhance the effects of the medication. Additionally, surgical treatments such as deep brain stimulation (DBS) are being studied for their potential to relieve symptoms of Parkinson’s^101,102^.

## Clinical features

Clinical features in PD are slowly developing, usually starting subtle and over time becoming more evident. It has been described as a 10-year period to fully express most of the clinical features.

**Figure 2:**
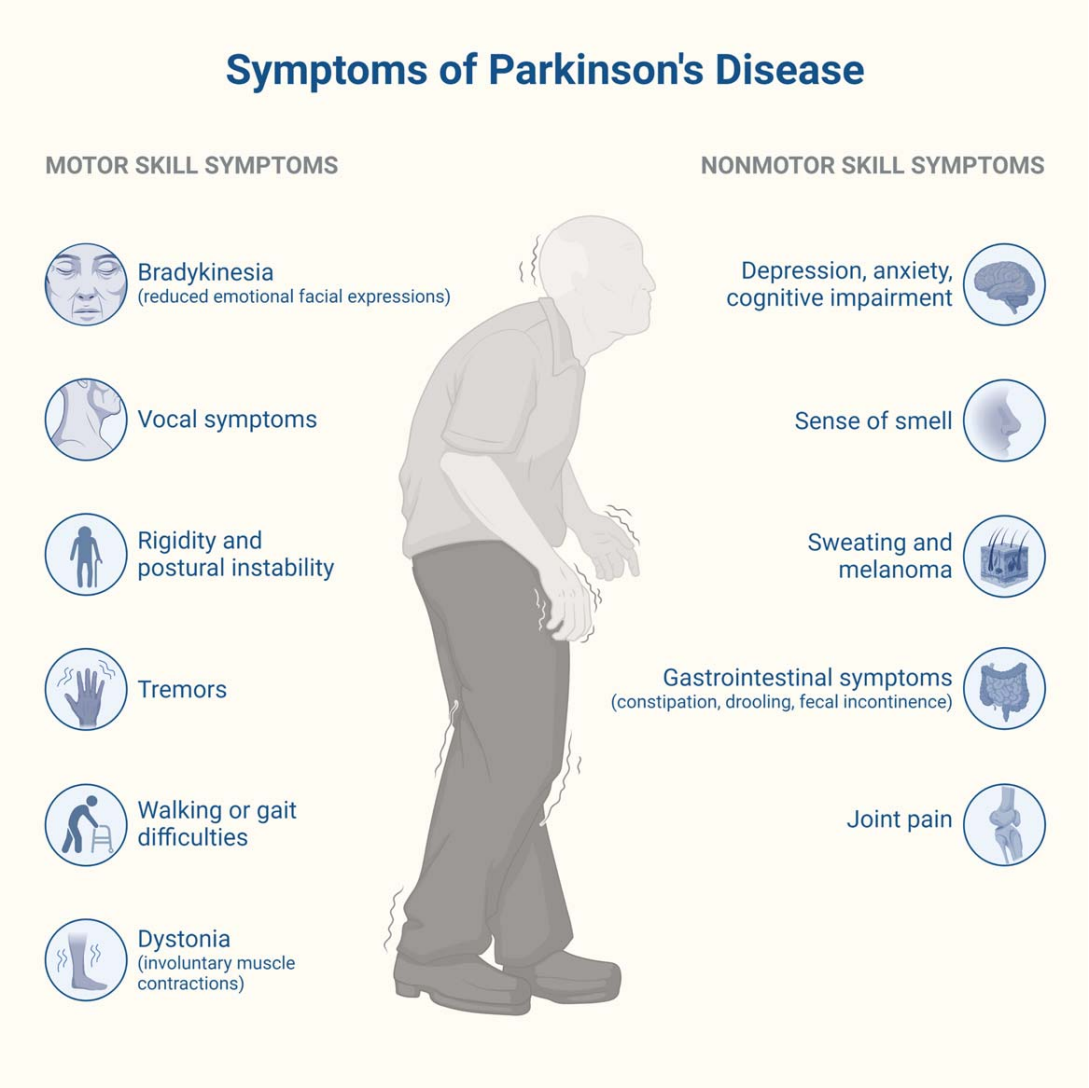
Symptoms of Parkinson’s disease.

Diagnosis is made by meeting the International Parkinson and Movement Disorder Society (MDS) criteria^103^ and described by the acronym *TRAP (tremor, rigidity, akinesia or bradykinesia, and postural instability)* (Fig. 2).

A resting tremor is seen when a limb is in a relaxed state, only applying force against gravity or resting on a surface, usually in a range of 4–6 Hz^103^. Its counterpart, essential tremor, has an amplitude of 8–10 Hz. Resting tremor is characteristically suppressed or diminished by movement and usually is the very first motor symptom in these patients.

The core feature, bradykinesia, is diminishing in speed, amplitude, or slow motion in movements made in a continuous manner, which becomes evident over time. It can also be present in voice and face (hypomimia). However, limb bradykinesia must be present to establish the diagnosis. The increased and persistent resistance to passive motion, specifically in the joints, is known as rigidity. This is different from spasticity because it is speed independent. In clinical practice, it may be difficult to establish differences but remembering that spasticity is only seen in rapid movements may help. Postural instability is usually seen in the advanced stages of the disease and is correlated with the severity of the disease. It is described as the tendency to fall and imbalance. It can be assessed with the pull test, where the physician stands behind the patient and lightly lifts the shoulders forward or backward. This maneuver puts to the test the ability to maintain balance^104^ and is positive when there is ineffective balance.

All the symptoms described above account for the ultimate diagnosis of parkinsonism. In addition to these symptoms, all other possible causes need to be excluded to continue confirming the diagnosis.

NMSs may also be present, which were first described two centuries ago, and acquired more relevance over the last two decades; these symptoms correlate clinically with the quality of life in patients and may appear up to a decade before the diagnosis is made. For instance, dementia may be seen in patients in a later stage of the disease. It has been reported in up to 50% of patients and is caused due to the imbalance between dopamine and acetylcholine in the central nervous system; therefore, a yearly screening is recommended for PD patients^105^.

Urinary dysfunction/incontinence is seen at any stage of PD, although the early presentation may be concerning for multiple system atrophy (MSA). NMS related to sleeping disturbances include excessive daytime sleepiness, sleep fragmentation, insomnia, REM sleep behavior disorder, restless legs, central sleep apnea, and nocturnal akinesia. These can occur at any disease stage^106^. These have a reported incidence of 75–80% in patients. These conjunctions of disturbances are not innocuous to the already altered brain, intermittent hypo-oxygenation in the brain, and inflammation may hasten the degenerative effect. Sleep apnea also correlates with cardiovascular morbidity.

### Orthostatic hypotension (OH) in PD

OH is a common NMS of PD, often referred to by the patients as a sensation of dizziness or even a fall. The criterion for diagnosis is a decrease of 20 mmHg in systolic blood pressure within 3 min of standing up or tilting upright or a decrease of 10 mmHg in diastolic blood pressure^107^.

Autonomic nervous system dysfunction is the main etiology of this symptom in this clinical scenario due to the pathologic accumulation of phosphorylated αSyn in the ganglia, neurons, or both. It is also caused due to decreased cerebral perfusion pressure. Remembering the normal translation and protein synthesis can help in the understanding of its biochemistry pathway disturbance. The folding of proteins begins in translation, and it is necessary for the protein to achieve a correct function in the organism or within the cell. Hydrophobic interactions^108^ and other proteins (such as chaperones)^109^ are the ones that regulate such processes and help in achieving a three-dimensional structure. The opposite mechanism which results in misfolding is the consequence of the hydrophobic amino acid residues on the surface of misfolded protein aggregation to other hydrophobic surfaces.

Peripheral vasoconstriction is impaired in PD^110^, which results in the inability to release norepinephrine and the fall in BP upon standing. All the mechanisms described previously can result in OH; however, daily activities such as eating may also exacerbate it^110^ by inducing an increase in blood pooling in the splanchnic bed (*postprandial hypotension*).

## Genetics

The study of genetics and the use of molecular markers in the identification of parkinsonism, a neurodegenerative disorder, has significantly advanced in recent years. Molecular markers are genetic sequences that give us a greater understanding of the molecular mechanisms which underlie the development, progression, and manifestation of certain diseases^111^. By using molecular markers to identify specific genetic changes in individuals suffering from PD, scientists have been able to develop better treatments and therapies. One of the earliest molecular markers of parkinsonism was discovered in 1997. Known as the *LRRK2* gene, this gene is associated with a higher incidence of PD compared to the normal population and has been found to be mutated in some patients. Other molecular markers of this disorder that have since been identified include the *PINK1* and the *PARK7* genes. While all three of these markers are known to be associated with the development of Parkinson’s, the *LRRK2* marker seems to be the most highly associated with the onset of the disorder^112–114^.

The neurogenic potential of a mutation in the *GBA* gene has also been confirmed by multiple studies. The same studies have identified other genetic variants in the same gene associated with PD, including haplotype and SNP (single nucleotide polymorphism) analysis. These studies have shown that mutations in the *GBA* gene increase the risk for PD and have a direct connection with other gene abnormalities, such as αSyn, that have been linked to PD. In addition to the *GBA* gene, several other genes associated with PD have been studied, including the *SNCA* gene. This gene contains the αSyn protein, which is important for maintaining the structure of neurons and the integrity of neuronal networks. Mutations in this gene can result in the buildup of αSyn in the brain, which has been linked to PD and other kinds of neurodegeneration. Gene silencing has also been utilized in studies to minimize αSyn expression and hence the likelihood of developing PD^115,116^.

Genetic testing has become increasingly useful for the diagnosis and management of PD. To date, genetic testing for PD has not been recommended for individuals with a high risk of developing the disease. However, the increasing knowledge gained from molecular markers of parkinsonism – along with other studies – points to a future in which genetic testing may have a role in the screening, diagnosis, and treatment of PD. Such advances in genetics will help us to better understand the disease process and its underlying causes, allowing us to develop more effective treatments and prevention strategies in the future^117^. Additionally, researchers have identified differences in the expression of certain genes as a possible predisposition for PD. These genes expression is believed to be linked to symptoms appearance such as tremors, slow movements, and impairment in cognition associated with Parkinson’s. By studying the expression of these genes, scientists have been able to develop therapies that target these genes and the underlying mechanisms which are thought to be responsible for the development of Parkinson’s^118^.

In addition to the genetic markers which have been identified, researchers have also developed testing methods that allow us to identify any specific changes in the genomes of individuals with PD. These tests are known as ‘polymorphisms’ and involve looking for any specific changes in the structure of an individual’s genome which may be associated with Parkinson’s. By identifying the specific changes in the genes of an individual with Parkinson’s, doctors can better tailor treatments to their needs^119^. By studying the genetic literature and using molecular markers, researchers hope to gain a better understanding of how Parkinson’s develops and progresses. This could potentially lead to improved treatments for patients as well as improved outcomes. In the future, molecular markers of Parkinson’s may even be used as predictive tests to identify those at risk or those who may be more likely to develop the disorder in the future. Overall, the use of molecular markers in the study and identification of PD has opened up a whole new field of exploration. With more research and a better understanding of the molecular mechanisms which cause this disorder, it is becoming increasingly possible to identify at-risk individuals and develop more rigorous treatments and therapies^120^.

## Diagnosis

Accurate diagnosis of PD remains a challenging issue in neurology. During the prodromal period, PD mostly presents with nonspecific NMSs, including hyposmia, constipation, possible RBD, depression, anxiety disorder, and cognitive impairment. Since these symptoms are widely seen in other pathologies, it is unlikely to diagnose PD based only on prodromal markers^40–48,121^.

Clinical diagnoses remain the main diagnostic tool for PD, and experts have shown their accuracy to be 83.9% (95% CI 69.7–92.6%)^122^.

To diagnose clinically probable PD, the following criteria should be satisfied:

1. There are no absolute exclusion criteria.
2. No more than two red flags with two or more counterbalancing supportive signs. If a patient has only one red flag, one supportive sign can be enough.

Basic clinical screening is based on identifying clinical features such as bradykinesia, rigidity, tremor, and gait alteration. Additional supportive criteria for diagnosing PD are:

1. Returning to a near-normal level of function with dopaminergic therapy
2. Marked worsening of symptoms with dose decreases
3. Levodopa-induced dyskinesia
4. Olfactory dysfunction: hyposmia^123^
5. Cardiac sympathetic denervation
6. Essential symptoms: bradykinesia, in combination with at least one of the following:

- Rest tremor
- Postural instability is not related to primary visual, vestibular, or cerebellar dysfunction with rigidity^124^.

During the later stage, PD can be associated with postural instability, freezing of gait, balance alteration, dysarthria, and dysphagia. Low clinical diagnostic accuracy was shown for the early stages of PD^125^.

Clinical diagnoses were correct only 26% of the time for patients who were not responsive to therapy and whose symptoms had recently developed. Therefore, neuropathologic confirmation of PD remains the gold standard diagnostic tool, with a sensitivity of 88% and specificity of 68%, and is commonly performed after autopsy.

A comprehensive review of the efficacy of acute demanding tests with levodopa and apomorphine was performed. Apomorphine had a sensitivity of 0.86 and acute levodopa had a sensitivity of 0.75, which was not superior to chronic levodopa treatment^126^. Despite this, because these tests are widely available, they may be useful for differential diagnosis in clinical practice^127^. In terms of imaging methods, specific changes in the brain’s neuroanatomy related to PD have not been shown^128^. However, structural MRI can be useful to rule out secondary causes of parkinsonism symptoms, which are commonly seen after infarcts, abscesses, tumors, normal pressure hydrocephalus (NPH), etc.

MSA is commonly associated with atrophy and hypointensity of the putamen, pontocerebellar, and cerebellar peduncles. Progressive supranuclear palsy (PSP) presents with midbrain atrophy, with the dilated third ventricle and superior cerebellar peduncle atrophy that can be seen. Corticobasal degeneration (CBD) is diagnosed with asymmetric parietal cortical atrophy in the MRI. During later stages, these imaging methods can be helpful for differential diagnosis, especially with MSA, PSP, and CBD^129^.

If the case is difficult, PET F-DOPA (positron emission tomography with fluorine-labeled dihydroxyphenylalanine) and SPECT DAT (single-photon emission computed tomography with dopamine transporter) can be useful^126^, but their usage is restricted by their availability and cost. On a SPECT scan, PD can be present with asymmetric reduction of striatal tracer binding and putaminal hypermetabolism, along with occipital and parietal hypometabolism on a PET scan.

Based on the above, it can be considered that during the last 25 years, the accuracy of diagnoses for the early stages of PD has slowly improved, and new guidelines and recommendations are necessary based on new research findings.

## Differential diagnosis

During the early stage, clinical manifestation commonly overlaps with atypical parkinsonism, such as PSP and MSA.

The first step in the differential diagnosis of each pathology is based on clinical signs and symptoms. In order to facilitate this process, specific diagnostic criteria have been introduced^103,129^.

Diagnosis of clinically established PD requires:

1. Absence of absolute exclusion criteria
2. Absence of red flags
3. At least two supportive criteria (discussed above)

Absolute exclusion criteria:

1. Cerebellar abnormality signs such as cerebellar gait, intention tremor, limb ataxia, etc. Cerebellar dysfunction can be similar to PD due to tremors and gait instability, and it should be ruled out first.
2. Supranuclear gaze palsy or slowing vertical saccades are commonly associated with PSP. PSP usually presents with early-onset postural instability and falls without losing consciousness, dysarthria, dysphagia, and marked frontal dementia.
3. Signs that satisfy the frontotemporal dementia behavioral variant (bvFD)^130^ or primary progressive aphasia (PPA).
4. Symptoms present only in lower limbs throughout 3 years or more. Commonly associated with secondary parkinsonism, especially vascular abnormalities^131^. Vascular parkinsonism (VaP) usually presents sudden onset bilateral lower extremity symptoms. Even though patients state that the dopaminergic therapy is not efficient, it might be helpful in some cases.
5. History of taking antipsychotic or dopamine-depleting medications, which is consistent with drug-induced parkinsonism (DIP). DIP presents with symmetric bradykinesia symptoms, with perioral tremor and tardive dyskinesia commonly associated. Discontinuation of the causative drug commonly leads to prompt improvement of symptoms. Other classes of drugs can lead to DIP as well, such as antiepileptics, antidepressants, calcium channel antagonists, etc.
6. Absence of improvement of symptoms even with a high dose of levodopa. Marked responses to levodopa therapy in 91% of cases were associated with PD. Although the absence of a response cannot rule out PD, most cases are associated with other conditions, commonly with PSP and CBD^132^.
7. Clear signs of dysfunction of the cortical region, such as apraxia, aphasia, graphesthesia, stereognosis, etc. Many causes lead to this kind of dysfunction. It can be localized, which is associated with tumors, stroke, abscess, trauma, seizures, etc. Or diffuse, commonly seen after hypoglycemia, hypoxia, infections, vasculitis, AD, etc.
8. Normal functioning dopaminergic system on neuroimaging. The function of the dopaminergic system can be assessed by dopamine transporter SPECT or meta-iodobenzylguanidine SPECT. On a fluorodeoxyglucose PET, PD is presented with putaminal hypermetabolism, while other parkinsonian causes are associated with hypometabolism signs.
9. If a patient has an alternative condition that can produce parkinsonian symptoms, it should be considered first and ruled out before suspecting PD.

## Pharmacologic therapy

The main purpose of PD treatment is symptomatic therapy. Unfortunately, currently, there are no disease-modifying (neuroprotective) treatment methods available.

Usually, dopaminergic therapy is recommended if a patient’s symptoms are severe enough and affect the quality of life, but it can also be used as a diagnostic tool in combination with other diagnostic methods. The medications need to be selected based on the patient’s complaints, symptoms, age, disease stage, and functional disabilities. In addition, the patient’s therapy needs to be corrected based on the progression of the symptoms.

Although most PD medications, including levodopa-based dopamine agonists and MAIs (monoamine oxidase inhibitors), are intended to treat all motor symptoms, they can be used for tremors. Amantadine can be used as an adjunctive therapy for treating tremor symptoms.

For dyskinesia symptoms, selegiline is used as an adjunctive therapy, and amantadine, propranolol, trihexyphenidyl, benztropine, or clozapine can be used as the main therapy.

For patients with severe symptoms, the initial therapy can be started with levodopa or dopamine agonists. For patients over 60 years, levodopa has shown more efficiency and fewer side effects like freezing, edema, and hallucination compared to dopamine agonists. By contrast, dopamine agonists have shown good relief of symptoms in patients younger than 60 years with mild symptoms. These medications showed a lower possibility of developing dyskinesia symptoms^133^.

### Consider adjunctive therapy

During treatment, ‘off’ periods may develop. To address this issue, the frequency of levodopa can be increased, or other classes of medicine can be added. A new extended-release levodopa-carbidopa formulation showed efficiency in reducing off-time compared to the immediate-release formulation^134^. Studies recommend using two classes of drugs: MAOBi (monoamine oxidase type B inhibitors) and entacapone^135^. Recent studies have shown that rasagiline (MAOBi) is more efficient than entacapone for addressing symptoms during the ‘off’ period^136^. Although rasagiline has shown good efficiency^137^, the efficiency of selegiline is ambiguous for reducing symptoms during the ‘off’ period^138^.

In terms of dopamine agonists, prolonged-release ropinirole has shown the best efficiency compared to other medications in its class^139^. A list of medications that are used for motor symptoms in PD are described in Table 2.

**Table 2:** List of medications with their class and side effects that are used for motor symptoms.

Main indication	Drug class	Medication’s name	Side effects
Severe motor symptoms	Levodopa derived:	Levodopa-carbidopa	Nausea and vomiting, constipation, depression, orthostatic hypotension, dyskinesia, and hallucinations
		Levodopa-benserazide	
	Dopamine agonist:	Pramipexole	Nausea and vomiting, somnolence, dyskinesia, orthostatic hypotension, hallucinations, ICDs, edema, and increased sleepiness (including sleep attacks)
		Ropinirole	
		Rotigotine	
Mild motor symptoms or fluctuations	MAOBIs	Selegiline	Nausea and vomiting, dyskinesia, abdominal pain, dry mouth, the stimulant effect, and exacerbation of levodopa adverse effects
		Rasagiline	EPS (dyskinesia, dystonia), headache, nausea and vomiting, orthostatic hypotension, arthralgia, dyspepsia, depression, flu-like syndrome, exacerbation of levodopa adverse effects, and constipation
		Safinamide	Nausea and vomiting, dyskinesia, hypertension, increasing liver enzymes, and insomnia
Motor fluctuations	COMTIs	Entacapone	Nausea and vomiting, dyskinesia, diarrhea, dark-colored urine, and exacerbation of levodopa adverse effects
		Tolcapone	Nausea and vomiting, dyskinesia, insomnia, dark-colored urine, hallucinations, headache, exacerbation of levodopa adverse effects, and hepatotoxicity
Gait instability and dyskinesia	Antiviral	Amantadine	Hallucinations, confusion, edema at the ankle, livedo reticularis, nausea, dry mouth, blurred vision, and constipation
	Antipsychotic	Clozapine	Hypersalivation, agranulocytosis, myocarditis, constipation, seizures, sedation, fever, dizziness, and orthostatic hypotension
Tremor	β-Blocker	Propranolol	Fatigue, bradycardia, hypotension, hypoglycemia, and depression
	Anticholinergic	Trihexyphenidyl	Hallucinations, rash, CI, nausea, dry mouth, blurred vision, urinary retention, and constipation
		Benztropine	

### Algorithm of treatment

If the first-line medication is not effective, the next class of drugs can be added or replaced (Table 3).

**Table 3:** Treatment algorithm for Parkinson’s disease symptoms.

Patients’ main concern	Mild symptoms	Severe symptoms
Tremor	Beta-blockers	Levodopa (first line if patient older than 60 years old)^152–154^
	Anticholinergic drugs	Clozapine^129,142,143^
		Surgical intervention
Bradykinesia and slowness	MAOBis	Age <60 years: dopamine agonists±levodopa^129,140,141^
		Age >60 years: levodopa ±dopamine agonist, COMTi, MAOBi^129,140,141^ Deep brain stimulation
Postural instability and gait impairment	MAOBi^144^	Age <60 years: dopamine agonists±levodopa^129,140,141^
	Amantadine^129,145,146^	Age >60 years: levodopa±amantadine, cholinesterase inhibitors If symptoms are fluctuating: levodopa±dopamine agonist, COMTi, MAOBi deep brain stimulation

## Monitoring for side effects and management of possible NMSs

A common side effect that can be seen during PD therapy is dyskinesia. Reducing the dosage of levodopa or dopamine agonist can rarely be considered, as it may worsen symptoms. For this purpose, amantadine is frequently used to reduce the severity and duration of dyskinesia.

Nausea symptoms commonly disturb patients who have recently started dopaminergic therapy or increased doses significantly. It can be reduced by titrating doses and slowly increasing medication dosage. In addition, administering food decreases the rate and amount of absorption of levodopa, which is also used to decrease side effects^147^. During severe nausea and vomiting, trimethobenzamide and domperidone have been recommended for use^148–150^. Other dopamine antagonists like metoclopramide should be avoided to prevent the worsening of parkinsonism symptoms.

### Psychosis

Before initiation of treatment for Parkinson’s disease psychosis, other causes related to systemic illness, metabolic disturbances, or psychiatric conditions needs to be addressed first or ruled out^151^.

The patient’s medication list needs to be reviewed to eliminate polypharmacy. Nonessential medications such as anticholinergics, benzodiazepines, sedating drugs, and steroids should be titrated to the lowest effective dose or stopped. Modification of levodopa’s dosage needs to be considered as a last option. Initial treatment usually starts with nonpharmacologic measures^152^, which include

1. Reestablishing circadian rhythm
2. Restoring sight and hearing
3. Environmental modification.

If these measures are not enough, antipsychotic therapy with clozapine and quetiapine can be considered. However, it can lead to the exacerbation of extrapyramidal symptoms. Although clozapine is more efficacious, quetiapine has been used as a first-line therapy because it does not require continuous monitoring and is less associated with side effects^153,154^.

### Dopamine dysregulation syndrome (DDS)

DDS is commonly seen during dopamine replacement therapy (DRT) in PD, with a prevalence of up to 4% in the population with movement disorders. Commonly characterized by addictive behavior to DRT, patients are increasing dosage despite improvement of motor symptoms and developing severe side effects such as dyskinesia. Mood changes, including mania, hypomania, depression, and dysphoria, are commonly seen after withdrawing from DRT. DDS is commonly implicated with levodopa and commonly seen together with other comorbidities such as psychosis, gambling, hypersexuality, punding, etc. The first and most successful treatment of these symptoms requires strict control of DRT.

Although there is no randomized controlled trial (RCT) available, some studies have shown the efficiency of valproic acid, which demonstrated complete resolutions of symptoms^155,156^.

### Rapid eye movement sleep behavior disorder (RBD)

Clonazepam is considered the first-line therapy for RBD^157–160^. Although the efficiency of melatonin and rivastigmine is not established, one RCT showed their beneficence^159–161^.

If the patient has an intolerance to clonazepam or contraindications, melatonin can be tried as a second-line therapy.

### Hallucinations

Hallucinations are a common manifestation of PD, with almost 70% of patients encountering them at least once during their life^161–163^. If hallucinations develop in patients with PD, it may be an indication of the severity of symptoms and usually develops in later stages and is commonly associated with cognitive impairment. If they develop acutely, other underlying causes should be ruled out, such as systemic illness, metabolic disturbances, other neurological and psychiatric causes, etc. Before adding new antipsychotic medication, optimization of the treatment plan should be considered^164^. Dopaminergic agonists commonly lead to side effects such as hallucinations with high doses, especially in the early stages. Cholinesterase inhibitors, especially rivastigmine, have shown good efficiency and relatively low side effects^165^. Pimavanserin can be an alternative option, which has good efficiency for parkinsonism-related hallucinations, but its usage can be limited due to unavailability throughout the world^166^.

### Mild cognitive impairment

For patients with mild cognitive impairment related to PD, atomoxetine showed a noticeable improvement in global cognitive performance^167,168^

### Parkinson disease dementia

The benefits of regular dementia therapy medications such as memantine, an *N*-methyl-d-aspartate receptor antagonist, are controversial^169^.

By contrast, one study showed improvement in verbal fluency and attention in patients with mild cognitive impairment by rasagiline^170^ despite its usage being limited due to the exacerbation of other potential symptoms such as tremors and gastrointestinal problems. Recent Cochrane studies showed moderate improvement in dementia, cognition, and behavioral symptoms by using rivastigmine^169–171^. On top of that, donepezil’s benefits are variable and require additional studies^171^.

### OH

OH as a part of autonomic dysfunction is commonly seen during the progressive stage of PD, but it can be consequently worsened with dopaminergic therapy. For mild symptoms, non-pharmacologic measures such as increasing salt and water intake, head elevation, compression stockings, fludrocortisone, or domperidone may be beneficial for it^172^.

Midodrine showed efficiency for neurogenic OHs, including the patient with other conditions^167,168,173,174^, pyridostigmine also can be beneficial^175^. Although previous studies were not able to prove the beneficence of yohimbine, a recent study showed significant improvement^176^. Indomethacin’s beneficence for symptom improvement was also confirmed^177^.

Domperidone, a peripheral D2 antagonist, can be useful in addressing the side effects of dopaminergic therapy, including nausea and OH. All of these antihypotensive drugs have their side effects, and titration doses can help address them.

### Sialorrhea

Several antimuscarinic drugs were investigated:

1. Atropine showed significant improvement, but its application is relative due to its severe side effects, such as delirium and hallucinations^178^.
2. Glycopyrrolate showed significant improvement in sialorrhea scores^179^.
3. Ipratropium bromide did not show any significant clinical improvement^180^.

Undoubtedly, injection of botulinum toxins A and B can be beneficial. Its efficiency was shown in several studies^181–183^. Potential severe side effects, including dysphagia, may limit its usage.

## Non-pharmacologic therapy

Nondrug therapies are used in all phases of PD. Their spectrum essentially includes various exercise therapies, psychological and neuropsychological interventions, as well as stereotactic interventions for neuromodulation. Physical training can add to pharmacotherapy to improve motor performance and may have the potential to positively influence the progression of the disease and prevent complications. There are symptoms such as postural instability or dysarthria in the later stages of the illness, for which nondrug therapeutic options are at the forefront^184^.

Despite the widespread use of exercise therapies in everyday clinical practice and an increasing number of clinical studies in this area, there is little scientifically based knowledge about which of the different treatment methods are indicated for which patients. It is possible that the patient’s individual inclinations, training intensity, and therapy adherence are more important than the decision for a specific form of therapy.

## Surgical management

The mainstay management of PD includes a combination of dopaminergic and non-dopaminergic options. Early medical therapy has been shown to be beneficial in reducing motor symptoms as well as life quality in the majority of individuals. Sadly, this is not always the case; when the illness progresses, and dopaminergic medications are continued, medical care may eventually fail to regulate the motor functioning of PD patients. In such cases, surgical alternatives should be considered.

Some of the advanced treatments currently available include DBS, dopaminergic infusion devices [levodopa-carbidopa intestinal gel (LCIG) infusion system], and lesioning procedures. Additionally, because there is no proof that these therapies may change the course of the disease, choosing such treatment options is heavily driven by the patient’s contentment with symptom management and ability to do assigned duties.

### DBS

One of the most prevalent surgical procedures to reduce the motor symptoms of PD is DBS. Not only that, but it also lessens the ‘off’ periods that occur along the day in more severe forms of medically managed PD. This technique involves delivering an electrical current through electrodes connected to a neurostimulator implanted surgically, resulting in the modification of neural networks. This is widely regarded as the most significant advancement in PD treatment since the advent of levodopa.

The globus pallidus interna (GPi) and the subthalamic nucleus (STN) are the most typically targeted brain areas by electrode implantation in PD. Many studies have investigated these locations and shown a comparable improvement in motor symptoms and quality of life^185–187^. Nonetheless, studies have shown that dopamine replacement drugs are reduced following subthalamic nucleus deep brain stimulation (STN-DBS), but GPi DBS has fewer cognitive and emotional adverse effects^186,187^.

DBS for GPi reduces levodopa-induced debilitating dyskinesias immediately and significantly. The influence on OFF symptoms may be less dramatic. The great decrease in dyskinesias, on the other hand, allows for a further rise in dopaminergic medication and subsequent improvement of motor symptoms.

Targeting the pedunculopontine nucleus may also assist in alleviating gait instability and freezing^188,189^.

### Lesioning procedures

Lesioning procedures function by targeting a specific brain tissue to disrupt the malfunctioning neuronal network. However, their use has been reduced since the introduction of levodopa first and DBS second. Radiofrequency thermal ablation, stereotactic ablative radiotherapy (SABR), magnetic resonance-guided focused ultrasound (MRgFUS), and MRI-guided laser interstitial thermal therapy (MRgLITT).

### Radiofrequency ablation

This has been one of the favorable methods for intracranial lesioning. It also has been implemented in the management of pain and epilepsy^190^. With this type of surgical intervention, the size of the lesion is dictated by the electrode size, the exposed tip length, and the temperature and duration of the lesion. For an effectively contained lesion, the size of the lesioning electrode is crucial. For example, a significant lesioning volume is required for GPi and anterior capsulotomy, demanding the use of a larger electrode.

During the procedure, the patient remains in a conscious state, and a test stimulation is performed to check the tissue of interest. An alternating current is directed at the tip of the active electrode inducing a thermal lesion. After that, the electrode is retracted. This allows for immediate results with distinct lesion borders, thus allowing intraoperative confirmation of symptom improvement.

Most pallidotomy side effects include visual field impairments, paresis, and cognitive deficiencies, which are usually temporary owing to perilesional edema^191^.

### Stereotactic radiosurgery lesioning

Using Computerized Treatment Planning (CTP) and Stereotactic Image-Guided Navigation (SIGN), a single ionizing radiation in high dose is directed at a confined brain volume. The problem is that the absence of intraoperative feedback, fluctuating in the size of the lesion, the lesion boundaries being poorly defined, and being subjected to ionizing radiation are some of the drawbacks of utilizing such a method^192^.

### Focused ultrasound thermal ablation

This procedure involves performing thermal ablation to an intracranial region using high-intensity ultrasound beams. The utilization of magnetic resonance thermography and MRI navigation in combination allows for accurate lesion targeting and monitoring. A set of transducers sends waves of ultrasound through the skull to a predetermined location within the brain. The benefits of this method include the absence of ionizing radiation, swift outcomes, and the ability to monitor progress using real-time MRI imaging.

## Conclusion

PD is indicated by the dopaminergic-producing substantia nigra neuronal loss, leading to motor symptoms and NMSs in the affected patients. The pathogenesis of PD is thought to be influenced by both environmental and genetic factors. PD may exhibit other diseases at the same time, such as normal pressure hydrocephalus, type 2 diabetes mellitus, multiple sclerosis, and Lewy body dementia which make the management more challenging. PD can result in dementia with Lewy bodies (DLB) or dementia brought on by the buildup of αSyn. Cognitive decline, behavioral and psychiatric symptoms, and protein aggregation formation in the brain are all characteristics that PDD and AD have in common. A prevalent diagnosis that raises the risk of dementia and cognitive decline is mixed dementia, which is characterized by the coexistence of both AD and PD disease. Shared genetic risk factors may also contribute to the overlap between PD and AD. Treatment with cholinesterase inhibitors can alleviate symptoms in both DLB and PDD. Management of PD relies on both pharmacological and non-pharmacological therapies. Surgical management is indicated in chronic cases.

## Limitations

No study is perfect and there are limitations that need to be considered and drafted. While this review identifies various risk factors associated with PD, it is important to note that the causal relationships between these factors and the development of PD are not always well-established. Confounding factors and reverse causality may also contribute to the associations reported. Secondly, the complexity and heterogeneity of PD make it challenging to fully capture all aspects of the disease in a single review. Additionally, the evidence supporting the effectiveness of non-pharmacologic therapies and surgical interventions is limited, and further research is needed to establish their optimal protocols, long-term outcomes, and potential complications. Lastly, the interpretation and generalizability of the findings may be influenced by variations in study designs, populations, and methodologies across the included literature, and there is a risk of publication bias.

## Ethical approval

Ethical approval is not required as it is a review article.

## Consent

None.

## Sources of funding

None.

## Author contribution

P.P.: conceptualization, visualization, and writing – original draft, review and editing; H.S.F.S. and K.A.: formal analysis, investigation, visualization, and writing – original draft, review and editing; T.T.: visualization, writing – original draft, review and editing, validation, and supervision; J.J., R.S.C.R., M.D.M.M., M.S., and A.A.: writing – original draft and review and editing; O.A.H.: writing – original draft, review and editing, and visualization.

## Conflicts of interest disclosure

The authors declare that they have no conflicts of interest.

## Research registration unique identifying number (UIN)

None.

## Guarantor

Herson S. Flores Sanga.

## Provenance and peer review

Not commissioned, externally peer-reviewed.

## Data availability statement

The data supporting the findings of this review are available within the review itself.
